# Correction to “The Prevalence, Distribution, and Clinicopathological Features of Seven Lung Cancer Actionable Driver Mutations in Taiwan”

**DOI:** 10.1111/1759-7714.70153

**Published:** 2025-08-26

Y.‐C. Lin, T.‐M. Yang, T.‐Y. Wang, et al., “The Prevalence, Distribution, and Clinicopathological Features of Seven Lung Cancer Actionable Driver Mutations in Taiwan,” *Thoracic Cancer* 16, no. 14 (2025): e70138, https://doi.org/10.1111/1759‐7714.70138.

In the Funding section and the Acknowledgments, the listed grant numbers were inconsistent and contained typos. The grants originally listed in the Funding section were CMRPG6F0471‐3, COPRG6K0011‐3, COPRG6K0041‐3, CORPG6K0031‐3. The grants listed in the Acknowledgments were COPRG6K0011‐3, CORPG6K0031‐3, CMRPG6F0471‐3, and CMRPG6N0151‐3. The correct grant numbers in both the Funding section and Acknowledgments should be CMRPG6F0471‐3, CORPG6K0011‐3, CORPG6K0041‐3, CORPG6K0031‐3, and CMRPG6N0151‐2.

We apologize for this error.

